# Predictors of pacemaker requirement in patients with implantable loop recorder and unexplained syncope: A systematic review and meta‐analysis

**DOI:** 10.1002/clc.24221

**Published:** 2024-01-29

**Authors:** Moein Zangiabadian, Kiarash Soltani, Yasaman Gholinejad, Reyhane Yahya, Shayan Bastami, Mohammad Ali Akbarzadeh, Mohammad Sharifian Ardestani, Azadeh Aletaha

**Affiliations:** ^1^ Endocrinology and Metabolism Re‐search Center, Institute of Basic and Clinical Physiology Sciences Kerman University of Medical Sciences Kerman Iran; ^2^ Shahid Beheshti University of Medical Sciences School of Medicine Tehran Iran; ^3^ Cardiovascular Research Center Shahid Beheshti University of Medical Sciences Tehran Iran; ^4^ Evidence Based Medicine Research Center, Endocrinology and Metabolism Clinical Sciences Institute Tehran University of medical Sciences Tehran Iran; ^5^ Endocrinology and Metabolism Clinical Sciences Institute, Endocrinology and Metabolism Research Center Tehran University of Medical Sciences Tehran Iran

**Keywords:** ILR, implantable loop recorder, pacemaker, predictor, unexplained syncope

## Abstract

Identifying the underlying cause of unexplained syncope is crucial for appropriate management of recurrent syncopal episodes. Implantable loop recorders (ILRs) have emerged as valuable diagnostic tools for monitoring patients with unexplained syncope. However, the predictors of pacemaker requirement in patients with ILR and unexplained syncope remain unclear. In this study, we shed light on these prognostic factors. PubMed/MEDLINE, EMBASE, Web of Science, and Cochrane CENTRAL were systematically searched until May 04, 2023. Studies that evaluated the predictors of pacemaker requirement in patients with implantable loop recorder and unexplained syncope were included. The “Quality In Prognosis Studies” appraisal tool was used for quality assessment. The pooled odds ratio (OR) with 95% confidence intervals (CIs) was calculated. The publication bias was evaluated using Egger's and Begg's tests. Ten studies (*n* = 4200) were included. Right bundle branch block (OR: 3.264; 95% CI: 1.907–5.588, *p* < .0001) and bifascicular block (OR: 2.969; 95% CI: 1.859–4.742, *p* < .0001) were the strongest predictors for pacemaker implantation. Pacemaker requirement was more than two times in patients with atrial fibrillation, sinus bradycardia and first degree AV block. Valvular heart disease, diabetes mellitus, and hypertension were also significantly more in patients with pacemaker implantation. Age (standardized mean difference [SMD]: 0.560; 95% CI: 0.410/0.710, *p* < .0001) and PR interval (SMD: 0.351; 95% CI: 0.150/0.553, *p* = .001) were significantly higher in patients with pacemaker requirement. Heart conduction disorders, atrial arrhythmias and underlying medical conditions are main predictors of pacemaker device implantation following loop recorder installation in unexplained syncopal patients.

## Graphical abstract

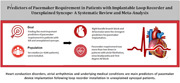

## 1 INTRODUCTION

Syncope is a kind of transient loss of consciousness due to cerebral hypoperfusion with spontaneous recovery.^1^ Based on statistics and reports, one of the common reasons of emergency visits is syncope with the incidence rate if 6.2 per 1000 person‐year and the recurrence rate of 21.6%.^2^ Interestingly, it has a two peak age distribution in younger patients around 20 years old and older ages around 70.^3^ The occurrence of syncope can affect the quality of life of individual with the fear of recurrence, trauma injury and social isolation. Besides, syncope have a socioeconomic burden with the annual cost of $1.7 billion for hospitalization.^4^ Syncope can be caused by different reasons such as cardiac issues, vasovagal reactions, orthostatic changes, medication side effects, transient ischemic attacks, and even cases of unknown origin.^2^ Among these causes, vasovagal syncope stands out as the most prevalent, accounting for at least 50% of the cases and occurrence of more than 33% in life time.^5^ Finding the underlying cause of syncope can be challenging due to the absence of the clinical presentation in the time of patients visits. Based on 2017 ACC/AHA/HRS Guideline for the Evaluation and Management of Patients with Syncope the initial evaluations of patients are history, physical examination and electrocardiogram (ECG).^6^ Besides the development of specialized investigation methods around 30% of patient's syncope remain unknown.^7^

Implantable loop recorder (ILR) is a cost‐effective small device that is implanted under the skin in order recording the electrical activity of the heart during a period of time. It is used in patients with unknown syncope who suspected with abnormal arrhythmic etiology.^8^ ILR is a potent diagnostic tool due to long term cardiac rhythm monitoring and the ability to detect approximately 50% of causes of syncope include bradycardia, asystole, atrioventricular block and ventricular tachycardia.^9^ Pacemaker therapy is one of the choice treatment strategies in certain causes of syncope like Sinus node dysfunction (SND), High‐degree atrioventricular block and Chronotropic incompetence which along with documented symptomatic bradycardia.^10^ Also, its benefit in vasovagal syncope is still controversial due to reasons such as infection, device malfunction and impotently in some studies they found no significant difference in outcomes between patients who received a pacemaker and those who did not.^11^

As a result, careful detection and selection of patients who advantage of pacemaker therapy can improve the prognosis and reducing the recurrence of unexplained syncope. There are some features such as age and clinical presentations include first degree atrioventricular block or PR interval which predicts, need of the implantation of pacemaker during ILR monitoring in patients with unexplained syncope.^12^

In this study we used a meta‐analysis to systemically investigate the predictive factors for pacemaker implantation to optimal and early management of patients with unexplained syncope.

## 2 METHOD

This study was performed and reported according to the Preferred Reporting Items for Systematic Reviews and Meta‐Analyses (PRISMA) 2020 statement.^13^ The study was registered in the Systematic Review Registration: PROSPERO (registration ID: CRD42023443796). According to our study design, ethics approval statement, and informed patient consent were not applicable.

### 2.1 Search strategy

We searched PubMed/Medline, EMBASE, Web of Science, and Cochrane CENTRAL for studies reporting the predictors of pacemaker requirement in patients with ILR and unexplained syncope, published up to May 04, 2023. Studies written in English were selected. We used the following MeSH terms: “‘Syncope’ and ‘Pacemaker, Artificial’.” Keyword searches were done with combinations of the terms “Syncope,” “Pacemaker,” “Implantable Loop Recorder,” “ILR,” “cardiac monitor*,” “cardiac device,” “loop device,” and “ICM” (Tables S1–S3). Backward and forward citation searching was performed.

### 2.2 Study selection

The records found through database searching were merged, and the duplicates were removed using EndNote X8 (Thomson Reuters). Two reviewers (KS and YG) independently screened the records by title/abstract and full texts according to the inclusion/exclusion criteria. Any disagreements were resolved by the lead investigator (MSA). Included studies met the following criteria: (i) patients with unexplained syncope who underwent ILR (ii) patients were divided in two groups on the basis of pacemaker requirement (pacemaker+ and pacemaker−) (iii) predictors of need to pacemaker that were reported between the two groups. Unexplained syncope was defined as a syncope that remained unexplained by the usual screening tests (history, clinical examination, orthostatic hypotension investigation, echocardiogram, ECG, and holter monitoring).^14^ The exclusion criteria were as follows: (i) evaluating pacemaker requirement predictors in nonunexplained syncope, (ii) assessing the predictors and outcomes of implantable cardioverter defibrillator in unexplained syncope, (iii) Comparing pacemaker and ILR outcomes. Also editorials, reviews, study protocols, meeting, or conference abstracts were excluded.

### 2.3 Data extraction

Two reviewers (SB, RY) designed a data extraction form. These reviewers collected data from all relevant studies, and disagreements were settled by consensus. The following data were extracted: first author name; year of publication; study design; countries where the research was conducted; demographics (i.e., age, sex); follow‐up time; Pacemaker implantation criteria; number of cases, controls and total population along by the predictors in each group.

### 2.4 Quality assessment

Two reviewers (SB, RY) assessed the quality of the studies using the Quality In Prognosis Studies (QUIPS) critical appraisal tool for studies of prognostic factors.^15^ If there were any discrepancies, a third reviewer was consulted. Items such as Study Participation, Study Attrition, Prognostic Factor Measurement, Outcome Measurement, Study Confounding, and Statistical Analysis and Reporting were evaluated. The traffic light plots and weighted bar plots for visualizing risk‐of‐bias assessments were designed with robvis tool.^16^

### 2.5 Statistical analysis

The pooled odds ratios (ORs) for dichotomous and standardized mean difference (SMD) for continuous data with 95% confidence interval (CI) were assessed using random or fixed‐effect models. The random effect model was used because estimated heterogeneity of the true effect sizes was high. The between‐study heterogeneity was assessed by Cochran's Q and the *I* ^2^ statistic. *I* ^2^ values more than 50% was considered as high heterogeneity.^17^ Meta‐analysis was done for predictors with at least three studies. The median and interquartile range were converted to mean and standard deviation for SMD calculation.^18^ Publication bias was evaluated statistically by using Egger's and Begg's tests (*p* < .05 was considered indicative of statistically significant publication bias).^19^ The funnel plot was not used for publication bias assessment because fewer than 10 studies were in each analysis.^20^ All analyses were conducted using “Comprehensive Meta‐Analysis” software, Version 2.0 (Biostat, Englewood, NJ).

## 3 RESULT

**Figure 1:**
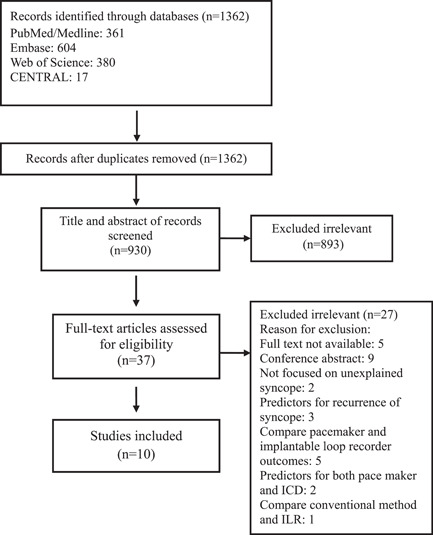
Flow chart of study selection for inclusion in the systematic review and meta‐analysis for predictors of pacemaker in unexplained syncope.

Figure 1 displays the flow diagram of study selection based on PRISMA. We identified 1362 papers through databases (PubMed/Medline, EMBASE, Web of Science, and Cochrane CENTRAL) and screened 930 papers after removing duplicates. First, we ruled out 893 papers by title and abstract since their subject or outcome were irrelevant to our study. We assessed 37 studies by full‐text review. Finally, 5 prospective^12^, ^21^, ^22^, ^23^, ^24^ and 5 retrospective^25^, ^26^, ^27^, ^28^, ^29^ observational studies were included.

### 3.1 Study and patient characteristics

As it shown in Table 1, four studies were conducted in Japan^22^, ^27^ and Spain.^23^, ^24^ Other article's origins were Canada,^21^ South Korea,^29^ Italy,^28^ Germany,^25^ Slovakia,^26^ and Australia.^12^ The follow‐up time ranged from 3 months to over 3 years. The total population who had unexplained syncope and ILR were 4200. The age range was 57–77.6 years (the mean age of total patients was 70.2 years old). Males were predominant with 2648 participants (63%). A total of 817 individuals (19.4%) underwent pacemaker implantation and others did not need pacemaker. Brady‐arrhythmic events were the most cause of pacemaker requirement.

**Table 1:** Study and patient characteristics.

First author	Publication year	Country	Study design	Follow up time	Pacemaker implantation criteria	Total population		Total age (mean)	
						Male (%)	Female (%)	Age pace (mean)	Age nonpace (mean)
Krahn et al.^21^	2002	Canada	Prospective observational	Minimum: 6 months	Bradycardia	216		57	
						124 (57.4)	92 (42.6)	62	56
Palmisano et al.^28^	2013	Italy	Retrospective observational	Every 3 months	Severe bradycardia (with heart rate b30 b.p.m.) and/or sinus arrest (>3 s), II‐ or III‐degree AV block with asystole >3 s and/or escape rhythm with a heart rate b30 b.p.m., Atrial fibrillation with slow ventricular response (b30 b.p.m.) and/or ventricular pauses >3 s	56		68.1	
						34 (60.7)	22 (39.3)	77.2	65.9
Mitro et al.^26^	2017	Slovakia	Retrospective observational	Mean: 233 days	Bradycardia less than 30/min, symptomatic or asymptomatic sinus pauses more than 3 s except for sleep, symptomatic AV block of 2nd degree and advanced AV block/complete AV block regardless of symptoms	112		64	
						49 (43.7)	63 (56.3)	68.1	60.7
Huemer et al.^25^	2017	Germany	Retrospective observational	Mean: 20 months	Sinus arrest, II‐ or III‐degree AV block, atrial fibrillation with a slow ventricular rate	106		59.1	
						50 (47.1)	56 (52.9)	62.1	58.3
Roca‐Luque et al.^24^	2018	Spain	Prospective observational	Mean: 27.9 months	Advanced AV block with or without syncope	159		74.1	
						97 (61.1)	62 (38.9)	74.8	72.8
Lee et al.^29^	2020	South Korea	Retrospective observational	3–6 months	A ventricular pause of >3 s, a ventricular rate of <40 beats/min, or a ventricular rate >180 beats/min for more than 16 beats.	173		67.6	
						107 (61.8)	66 (38.2)	74.5	64.2
Miyazaki et al.^22^	2022	Japan	Prospective observational	Median: 279 days	Detection of one or more Brady arrhythmic events (with or without syncope recurrence)	128		65.2	
						72 (56.2)	56 (43.8)	72.5	59
Xiao et al.^12^	2022	Australia	Prospective observational	Over 3 years	Sinus node dysfunction/sick sinus syndrome, high‐grade AV block	100		65.5	
						61 (61)	39 (39)	70.8	60.2
Francisco‐Pascual et al.^23^	2022	Spain	Prospective observational	Over 3 years	Bradycardia	245		77.6	
						144 (58.7)	101 (41.3)	Not available	Not available
Tonegawa‐Kuji et al.^27^	2023	Japan	Retrospective observational	Median: 128 days	Bradyarrhythmia (with or without a syncopal recurrence)	2905		71.6	
						1910 (65.7)	995 (34.3)	74.6	68.6

### 3.2 Quality of included studies

**Figure 2:**
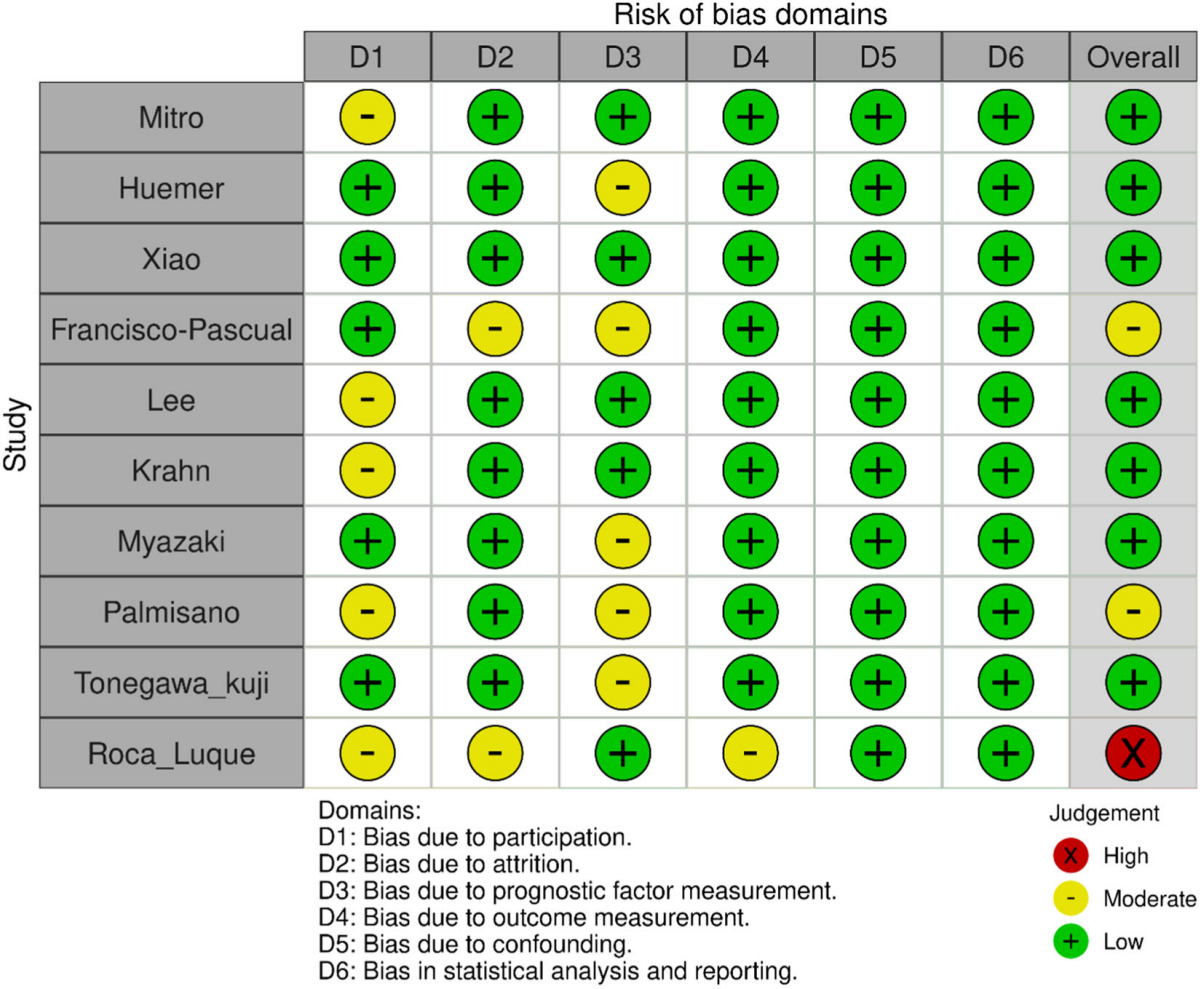
Traffic light plot of the domain‐level judgements for each individual result.

According to QUIPS critical appraisal tool,^15^ all included studies had low or moderate risk of bias except Roca‐Luque et al.^24^ study that was high risk in regard of participation, attrition and outcome measurement domains (Figure 2 and Figure S11).

### 3.3 Predictors of pacemaker implantation

The meta‐analysis of predictors with dichotomous data (Table 2) showed that pacemaker implantation in patients with unexplained syncope and ILR was about three times more in individuals with right bundle branch block (RBBB) (OR: 3.264; 95% CI: 1.907–5.588, *p* < .0001) and bifascicular block (OR: 2.969; 95% CI: 1.859–4.742, *p* < .0001). Sinus bradycardia (OR: 2.596; 95% CI: 1.072–6.289, *p* = .035) and first degree AV block (OR: 2.321; 95% CI: 1.350–3.990, *p* = .002) were the third and fourth strong predictors for pacemaker implantation, respectively. As it shown in Table 2, atrial fibrillation (AF), valvular heart disease, diabetes mellitus (DM) and hypertension (HTN) were significantly more in patients with pacemaker requirement. On the other hand, history of stroke, cardiomyopathy, ischemic heart disease, left bundle branch block and sex (male) were not significant predictors. Age (SMD: 0.560; 95% CI: 0.410/0.710, *p* < .0001) and PR interval (SMD: 0.351; 95% CI: 0.150/0.553, *p* = .001) were significantly higher in patients with pacemaker requirement than individuals without need to pacemaker but there was not significant difference for mean of body mass index and heart rate (Table 3). There was no evidence of publication bias (*p* > .05). Forest plots of the significant analyses are included as Figures S1–S10. Additionally, all available data on the duration of ILR implantation and the recorded findings are given in the Table S1.

**Table 2:** Predictors with dichotomous data.

Predictor	No. of study	No. of patients	Odds ratio (95% CI)/(*p*‐value)	Heterogeneity *I* ^2^ (%)/*p*‐value	Begg/Egger test *p*‐value
RBBB	6	3468	3.264 (1.907–5.588)/0.000	1.812/0.405	1.000/0.440
Bifascicular block	4	3365	2.969 (1.859–4.742)/0.000	0.000/0.742	1.000/0.895
Sinus bradycardia	3	341	2.596 (1.072–6.289)/0.035	39.527/0.191	1.000/0.733
First degree AV block	5	3352	2.321 (1.350–3.990)/0.002	0.851/0.401	0.806/0.417
AF	7	3580	2.194 (1.425–3.377)/0.000	42.485/0.108	0.763/0.399
Valvular heart disease	3	3134	1.792 (1.141–2.813)/0.011	0.000/0.805	1.000/0.709
DM	5	3368	1.630 (1.284–2.069)/0.000	0.000/0.853	0.220/0.348
HTN	6	3468	1.527 (1.135–2.175)/0.007	30.494/0.207	0.707/0.051
Cardiomyopathy	4	3240	0.602 (0.359–1.011)/0.055	0.000/0.947	1.000/0.906
IHD	6	3527	1.284 (0.947–1.741)/0.108	0.000/0.759	1.000/0.583
Male	8	1136	1.245 (0.945–1.641)/0.119	0.000/0.592	0.710/0.491
History of stroke	3	3089	1.020 (0.614–1.696)/0.939	0.000/0.900	1.000/0.584
NSVT	3	3089	0.602 (0.306–1.186)/0.142	0.000/0.446	1.000/0.803
LBBB	6	3499	1.903 (0.737–4.914)/0.184	53.264/0.058	1.000/0.733

Abbreviations: AF, atrial fibrillation; CI, confidence interval; DM, diabetes mellitus; HTN, hypertension; IHD, ischemic heart disease; LBBB, left bundle branch block; RBBB, right bundle branch block.

**Table 3:** Predictors with continuous data.

Predictor	No. of study	No. of patients	Standard mean difference (95% CI)/(*p*‐value)	Heterogeneity *I* ^2^ (%)/*p*‐value	Begg/Egger test *p*‐value
Age (year)	8	3796	0.560 (0.410/0.710)/0.000	37.824/0.128	0.710/0.064
PR interval (ms)	4	493	0.351 (0.150/0.553)/0.001	0.000/0.595	1.000/0.812
BMI (kg/m^2^)	3	407	0.289 (−0.283/0.860)/0.322	84.180/0.002	0.296/0.230
HR (b.p.m)	3	334	−0.120 (−0.359/0.120)/0.328	0.000/0.877	1.000/0.058

Abbreviations: BMI, body mass index; CI, confidence interval; HR, heart rate.

## 4 DISCUSSION

To the best of our knowledge, this study is the first systematic review to report the predictors of pacemaker implantation in patients with unexplained syncope receiving ILRs. ILRs assist clinicians in determining the cause of unexplained syncope, although predictors and the likelihood that a pacemaker would be needed later in the course of the disease are still up for debate.^27^, ^30^, ^31^ Recent studies have shown that pacemaker implantation may be beneficial for individuals with unexplained syncope who have an underlying arrhythmic etiology, alleviating syncope related symptoms such as bradyarrhythmia.^29^ Thus, early detection of such patients who benefit from this therapeutic approach can improve their overall prognosis.

RBBB and bifascicular block are two significant predictors of pacemaker implantation, and patients with unexplained syncope who also have RBBB and bifascicular block are three times more likely to need pacemaker implantation after ILR installation, according to our meta‐analysis of dichotomous and continuous data. Additionally, sinus bradycardia, first degree AV block, and AF are linked to a twofold increase in the probability of pacemaker implantation. Valvular heart disease, DM, HTN, age, and extended PR interval are also linked to pacemaker implantation, but to a lower extent.

RBBB and bifascicular block predispose patients to syncope of unknown origin mainly through induction of transitory conduction perturbations that are mostly caused at the atrioventricular (AV) node level.^32^, ^33^ Simple and precise detection of AV node block via ILR monitoring is the primary reason for pacemaker implantation in patients with unexplained syncope.^34^, ^35^ In a retrospective cohort study, 323 patients with at least one syncope and a history of BBB in the previous 6 months were included and were retrospectively followed for approximately 3 years; the authors came to the conclusion that in syncopal patients with a history of BBB, the insertion of an ILR achieves a high rate of etiological diagnosis, however the efficacy of pacing device installation remained debated.^36^ Consistent with our findings, in the SPRITELY trial, it was found that older patients with bifascicular block who received empiric permanent pacing therapy rather than ILR monitoring had a better prognosis.^37^

AF is an independent risk factor for the development of syncope and is associated with SND and sick sinus syndrome.^38^, ^39^ Following elongated intermittent AF pauses, syncope is more likely to occur in SND patients. In addition, AF associated tachycardia suppresses sinus node automaticity upon termination of tachycardia which results in sinus pauses with irregular duration.^27^, ^40^ From a histological standpoint, fibrosis and degenerative alterations at sinus node, atrium, and bundle branches are more frequently witnessed in patients with bradycardia‐tachycardia syndrome (so‐called sick sinus syndrome).^41^, ^42^ These modifications, particularly those near the atrium tissue, inhibit sinus node impulsion activity which is manifested as a prolonged PR interval in the EKG.^41^ Previous clinical studies demonstrate that abnormalities at atrium level namely AF, first degree atrioventricular block, and elongated PR interval increase the risk of recurrent syncope with the need for pacing device implantation which is concurrent with our result.^12^, ^22^, ^26^, ^27^

Sinus bradycardia was found to be a major predictive factor of pacing requirement based on our analysis which is the most common arrhythmia seen in individuals with unexplained syncope during ILR monitoring. Similar to other atrial dysfunction related complications, based on clinical and observational studies, sinus bradycardia is affiliated with reduction of heart rate mostly through SND or disturbances in cardiac conduction system which necessitates the need for pacemaker implantation. In earlier research done in this area, sinus bradycardia was the most prevalent arrhythmia found; for instance, in a study using data from the PICTURE registry in nearly half of the patients with diagnosed syncope, bradycardia was present.^31^ Additionally, a number of clinical studies identified sinus bradycardia as a predictive factor of pacemaker implantation which is in line with our results.^28^, ^43^, ^44^, ^45^

Valvular heart disease is another risk factor for the implantation of pacing device based on our study. Cardiac valvular disease causes structural alterations in cardiac muscles which decreases cardiac output.^27^, ^46^ Although structural heart disorders are known to increase the incidence of cardiogenic syncope, prior investigations have not consistently identified these structural abnormalities as risk factors for pacemaker implantation.^47^ This finding can be explained by the fact that, even while structural changes can result in cardiogenic syncope, the cardiac conduction system typically remains intact and the insertion of a pacemaker is not recommended in the absence of arrhythmia.^27^

Senility is another independent predictor of PDI, concurrent with previously conducted studies.^27^, ^28^, ^43^ Elderly patients are more likely to develop bradycardia with the need of pacing device implantation due to the age‐related degenerative changes, most notably progressive fibrosis, in cardiac conduction system.^43^

Diabetes was found to be a trivial predictive factor based of our results, which has been rarely reported as an outcome before our study.^27^ The exact underlying mechanism of syncope in diabetic patients is not thoroughly understood however it is believed that presence of autonomic neuropathy in diabetic individuals reduces sympathetic functions, which are necessary to maintain sufficient cerebral blood flow and make diabetic patients more susceptible to bradycardia‐associated syncope.^27^, ^48^, ^49^

HTN, similar to diabetes was found to be an insignificant independent predictive factor for pacemaker implantation. Only a few studies have identified HTN as a risk factor for the implantation of pacing devices, however HTN was more prevalent among patients that required pacemaker implantation according to data from other studies.^12^, ^23^, ^25^, ^26^, ^29^, ^50^ it's important to note that, based on the SPRINT trial, among hypertensive patients, aggressive HTN treatment compared to standard treatment was more associated with the occurrence of syncope.^51^ For the best care of syncopal patients, a precise understanding of the underlying mechanisms linked with syncope would be helpful given the high prevalence of high blood pressure, especially among older populations.

## 5 LIMITATION

Before interpreting the findings, it is important to be aware of various limitations in the current meta‐analysis. (i) Every study that is included was conducted in developed nations in Europe, North America, and East Asia. Therefore, studies from other regions should also be analyzed to reach a comprehensive result. (ii) The external validity of our investigation is constrained by the overall low number of included studies and the combined study populations across all included studies. (iii) Although we performed backwards and forward citation searching and hand searching for grey literature, searching Google Scholar and some other online databases like Scopus was not done. Therefore, we cannot rule out the possibility of not finding some relevant articles. (iv) All of the included studies are observational and provide no causal relationship between predictors and pacemaker implantation. (v) All non‐English articles were excluded that can lead to bias in the results.

## 6 CONCLUSION

According to our study, RBBB and Bifascicular block are significant predictors of pacemaker device implantation following loop recorder installation in patients with unexplained syncope episodes. Atrial related morbidities such as sinus bradycardia, first degree AV block, and AF are also highly associated with pacing device requirement. Other risk factors identified in our study are valvular heart disease, DM, and history of HTN. Further research and investigation concerning the underlying mechanisms involved in the pathophysiology of syncope are required to improve our understanding of the aforementioned factors.

## AUTHOR CONTRIBUTIONS

**Moein Zangiabadian and Mohammad Sharifian Ardestani:** conceptualized the topic. **Moein Zangiabadian:** searched the databases. **Yasaman Gholinejad and Kiarash Soltani:** performed screening and full‐text review. **Reyhane Yahya and Shayan Bastami:** performed data extraction. **Reyhane Yahya and Shayan Bastami:** performed quality assessment. **Moein Zangiabadian, Yasaman Gholinejad, and Kiarash Soltani:** prepared the first draft of the manuscript. **Mohammad Ali Akbarzadeh and Azadeh Aletaha:** critically revised and edited the manuscript. **Mohammad Sharifian Ardestani:** supervised this project. All authors reviewed and approved the final version of the manuscript.

## CONFLICT OF INTEREST STATEMENT

The authors declare no conflicts of interest.

## Supporting information

Supporting information.

Supporting information.

Supporting information.

## Data Availability

The data that support the findings of this study are available from the corresponding author upon reasonable request.
