# Supplementary material for: Predictors of pacemaker requirement in patients with implantable loop recorder and unexplained syncope: A systematic review and meta‐analysis
Source: Clin Cardiol. 2024 Jan 29;47(2):e24221. doi: 10.1002/clc.24221 (PMC10823547; doi:10.1002/clc.24221)
Supplement: Supplementary file 1 — Supporting information. [file CLC-47-e24221-s002.docx]

| #1 | (Syncope[MeSH Terms]) OR (Syncope[Title/Abstract]) |
| --- | --- |
| #2 | (Pacemaker, Artificial[MeSH Terms]) OR (pacemaker[Title/Abstract]) |
| #3 | ((((((implantable[Title/Abstract]) OR ("loop recorder"[Title/Abstract])) OR ("cardiac monitor*"[Title/Abstract])) OR ("cardiac device"[Title/Abstract])) OR (ILR[Title/Abstract])) OR (ICM[Title/Abstract])) OR ("loop device"[Title/Abstract]) |
| #4 | (((Syncope[MeSH Terms]) OR (Syncope[Title/Abstract])) AND ((Pacemaker, Artificial[MeSH Terms]) OR (pacemaker[Title/Abstract]))) AND (((((((implantable[Title/Abstract]) OR ("loop recorder"[Title/Abstract])) OR ("cardiac monitor*"[Title/Abstract])) OR ("cardiac device"[Title/Abstract])) OR (ILR[Title/Abstract])) OR (ICM[Title/Abstract])) OR ("loop device"[Title/Abstract])) |

Table S1. Search strategy of PubMed/Medline 4/5/2023

| #1 | 'cardiac rhythm management device'/exp OR 'artificial heart pacemaker'/exp OR pacemaker:ti,ab,kw |
| --- | --- |
| #2 | 'faintness'/exp OR syncope:ti,ab,kw |
| #3 | 'implantable cardiac monitor'/exp OR 'loop recorder':ti,ab,kw OR ilr:ti,ab,kw OR 'loop device':ti,ab,kw |
| #4 | ('cardiac rhythm management device'/exp OR 'artificial heart pacemaker'/exp OR pacemaker:ti,ab,kw) AND ('faintness'/exp OR syncope:ti,ab,kw) AND 'implantable cardiac monitor'/exp OR 'loop recorder':ti,ab,kw OR ilr:ti,ab,kw OR 'loop device':ti,ab,kw |

Table S2. Search strategy of Embase

| #1 | (TS=(Syncope)) OR TS=(faintness) |
| --- | --- |
| #2 | ((TS=(pacemaker)) OR TS=("cardiac rhythm management device")) OR TS=("artificial heart pacemaker") |
| #3 | ((((((TS=("loop recorder")) OR TS=(implantable)) OR TS=("cardiac monitor*")) OR TS=("cardiac device")) OR TS=("loop device")) OR TS=(ILR)) OR TS=(ICM) |
| #4 | (TS=(Syncope)) OR TS=(faintness) AND ((TS=(pacemaker)) OR TS=("cardiac rhythm management device")) OR TS=("artificial heart pacemaker") AND ((((((TS=("loop recorder")) OR TS=(implantable)) OR TS=("cardiac monitor*")) OR TS=("cardiac device")) OR TS=("loop device")) OR TS=(ILR)) OR TS=(ICM) |

Table S3. Search strategy of Web of Science

| #1 | MeSH descriptor: [Syncope] explode all trees |
| --- | --- |
| #2 | MeSH descriptor: [Pacemaker, Artificial] explode all trees |
| #3 | (pacemaker):ti,ab,kw |
| #4 | #2 OR #3 |
| #5 | (implantable):ti,ab,kw OR ("loop recorder"):ti,ab,kw OR ("cardiac monitor*"):ti,ab,kw OR (ILR):ti,ab,kw OR (ICM):ti,ab,kw |
| #6 | ("cardiac device"):ti,ab,kw OR ("loop device"):ti,ab,kw |
| #7 | #5 OR #6 |
| #8 | #1 AND #4 AND #7 |

Table S4. CENTRAL
