# Supplementary material for: Predictors of pacemaker requirement in patients with implantable loop recorder and unexplained syncope: A systematic review and meta‐analysis
Source: Clin Cardiol. 2024 Jan 29;47(2):e24221. doi: 10.1002/clc.24221 (PMC10823547; doi:10.1002/clc.24221)
Supplement: Supplementary file 2 — Supporting information. [file CLC-47-e24221-s003.docx]

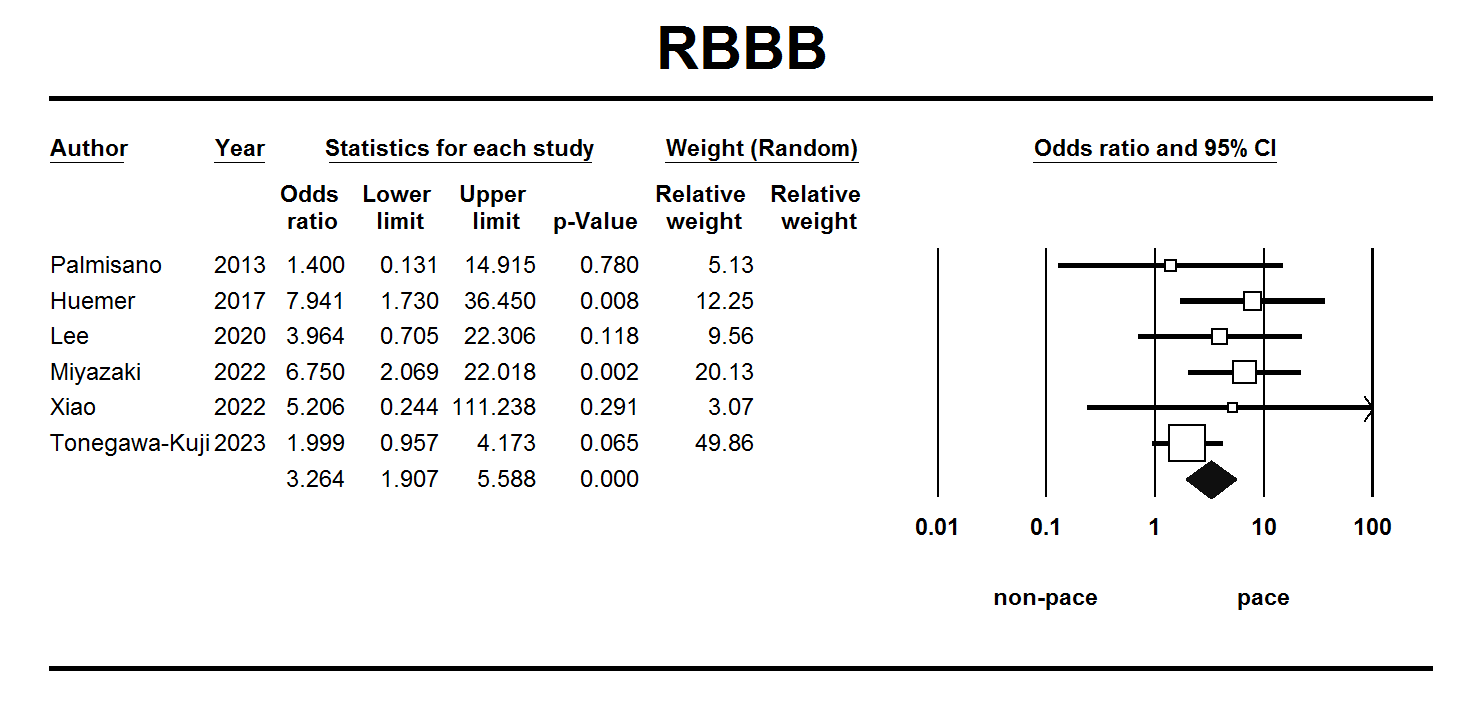


Supplementary figure 1. RBBB as predictor of pacemaker implantation in patients with ILR and unexplained syncope.


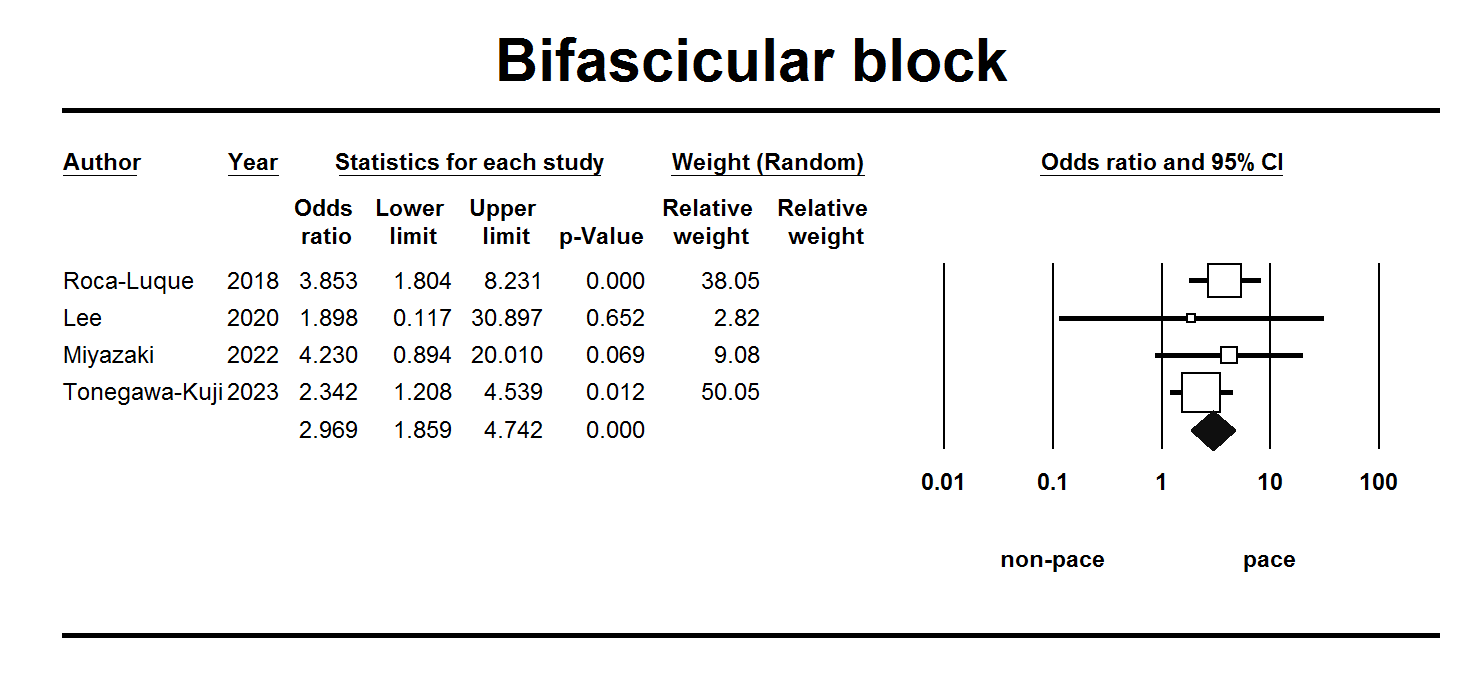


Supplementary figure 2. Bifascicular block as predictor of pacemaker implantation in patients with ILR and unexplained syncope.


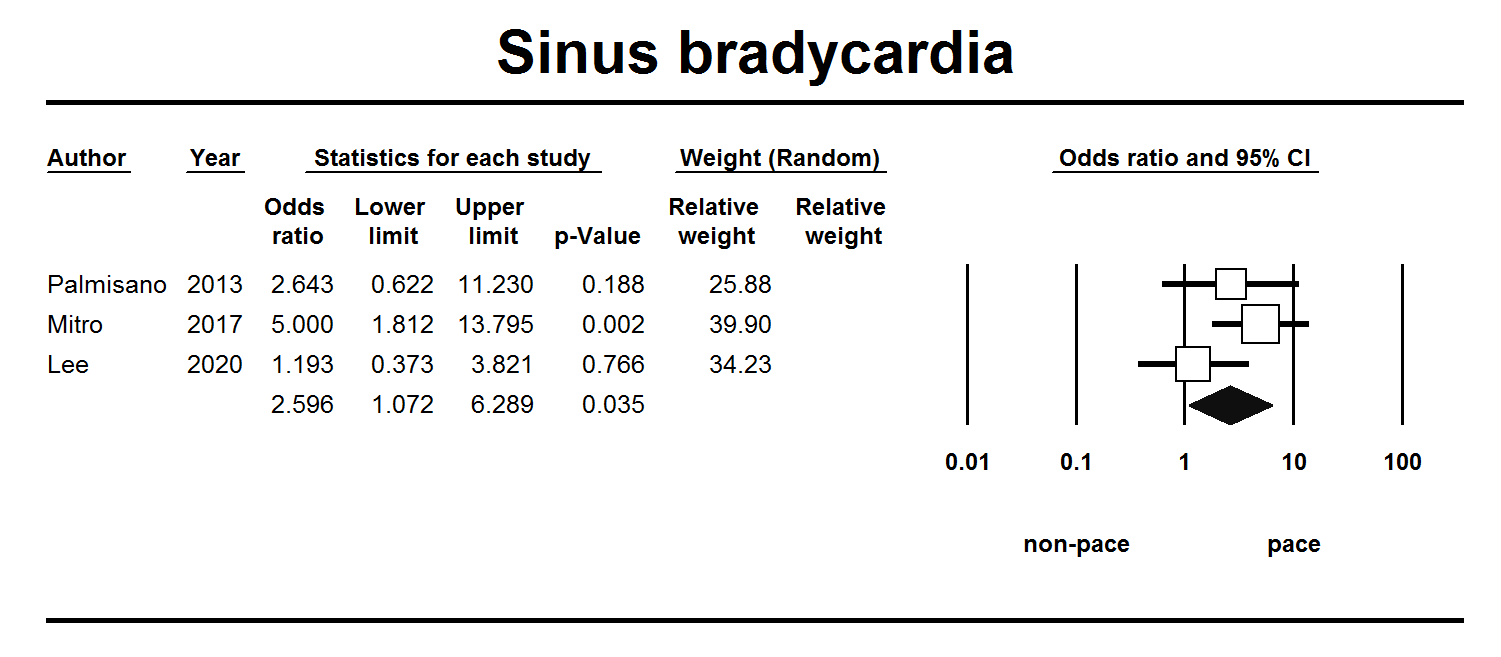


Supplementary figure 3. Sinus bradycardia as predictor of pacemaker implantation in patients with ILR and unexplained syncope.


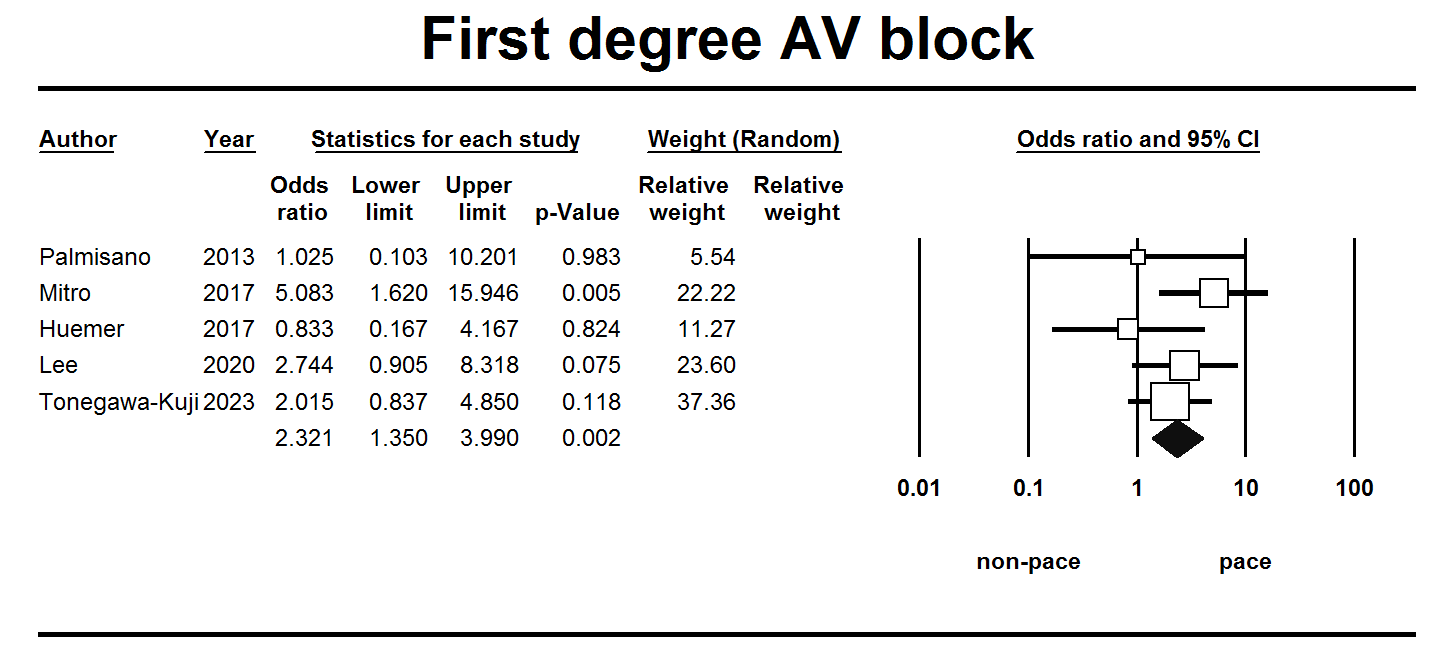


Supplementary figure 4. First degree AV as predictor of pacemaker implantation in patients with ILR and unexplained syncope.


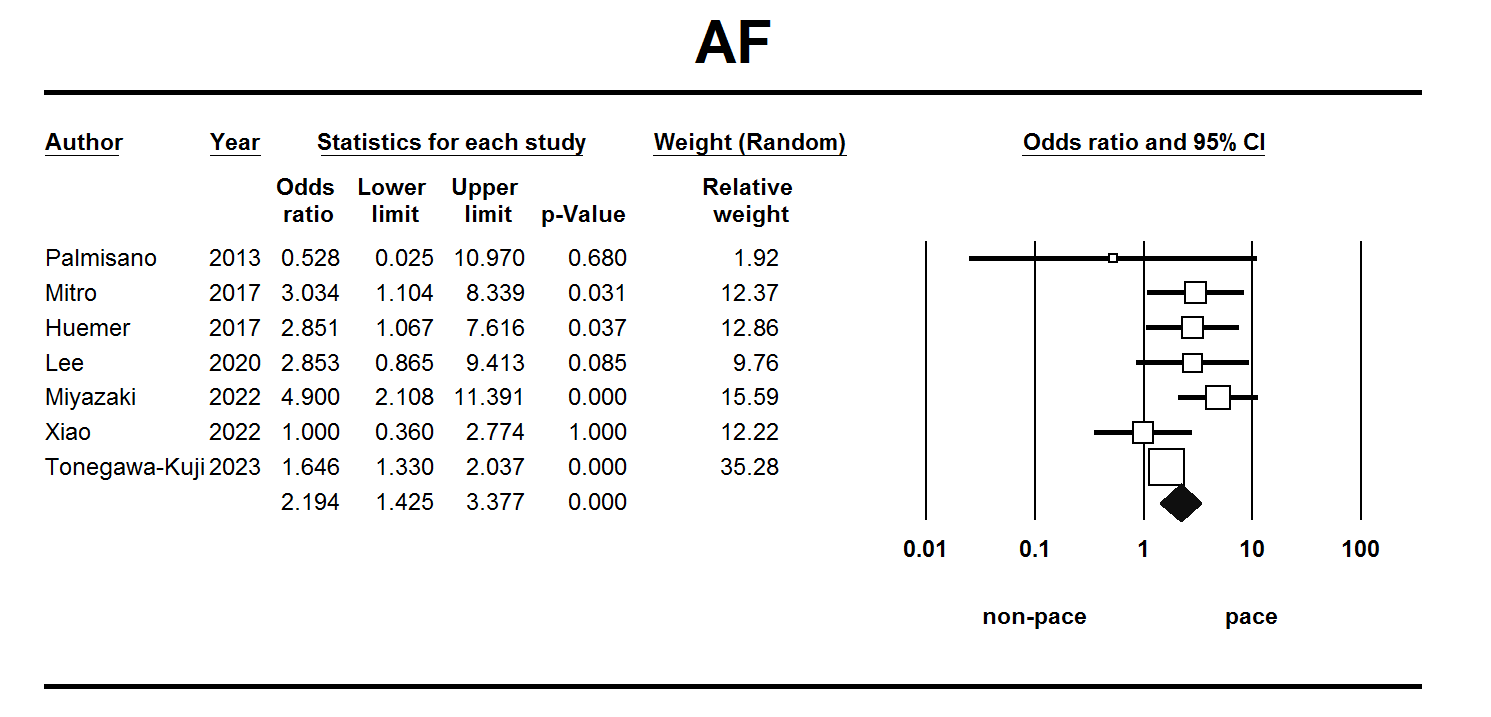


Supplementary figure 5. AF as predictor of pacemaker implantation in patients with ILR and unexplained syncope.


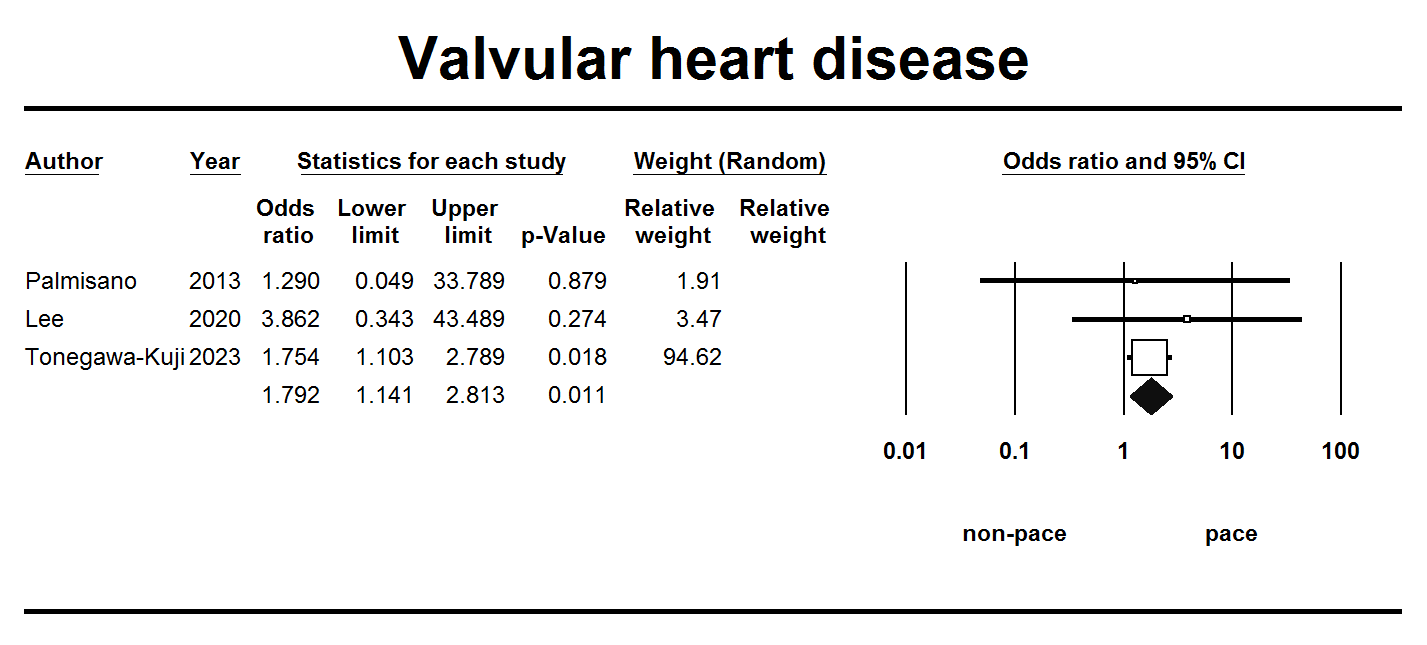


Supplementary figure 6. Valvular heart disease as predictor of pacemaker implantation in patients with ILR and unexplained syncope.


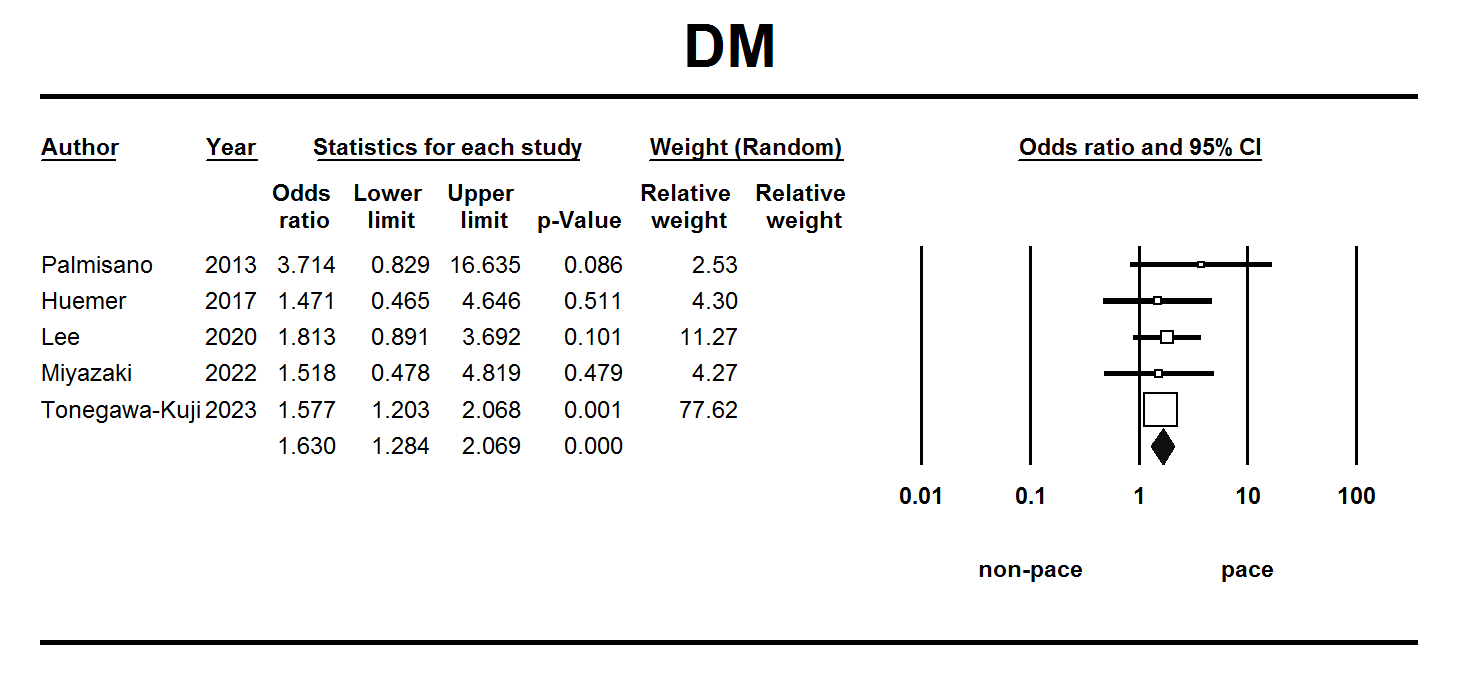


Supplementary figure 7. DM as predictor of pacemaker implantation in patients with ILR and unexplained syncope.


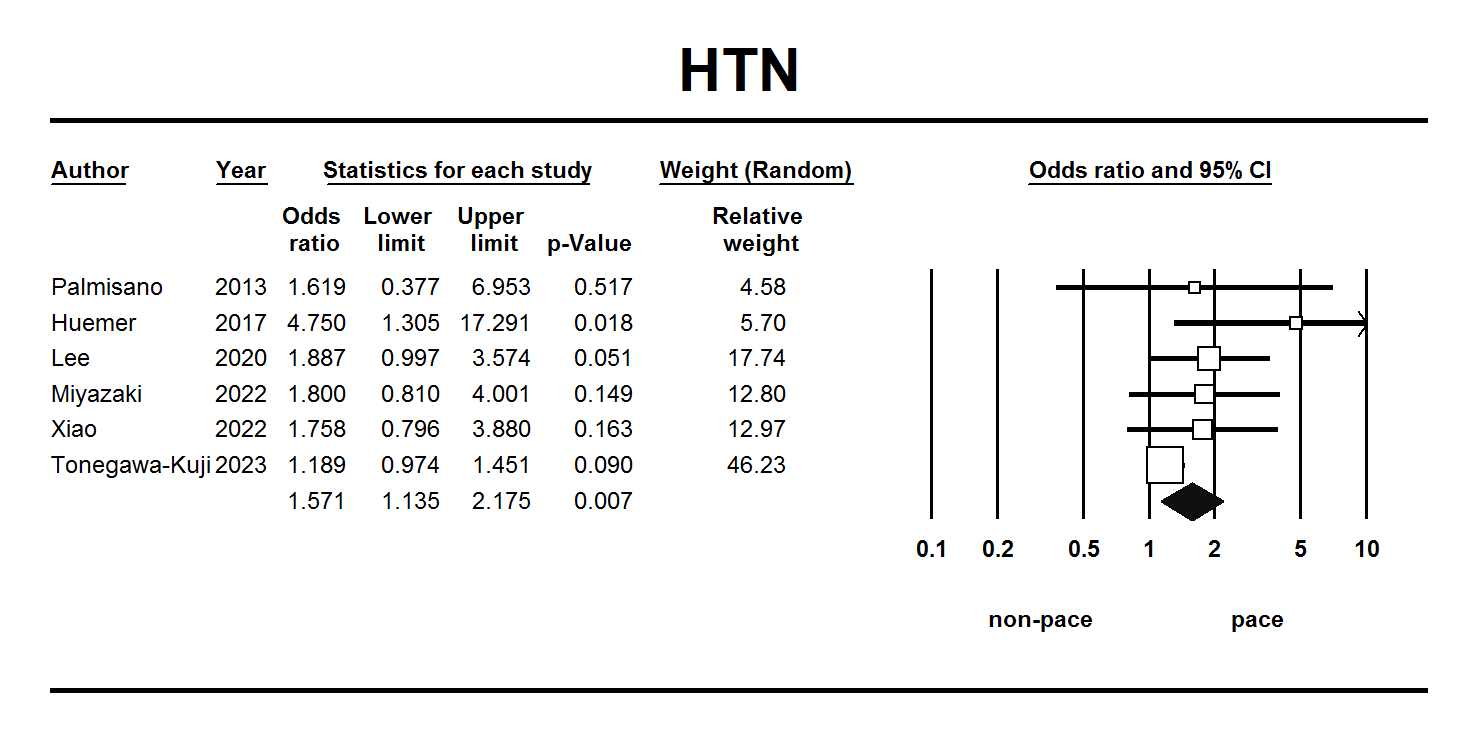


Supplementary figure 8. HTN as predictor of pacemaker implantation in patients with ILR and unexplained syncope.


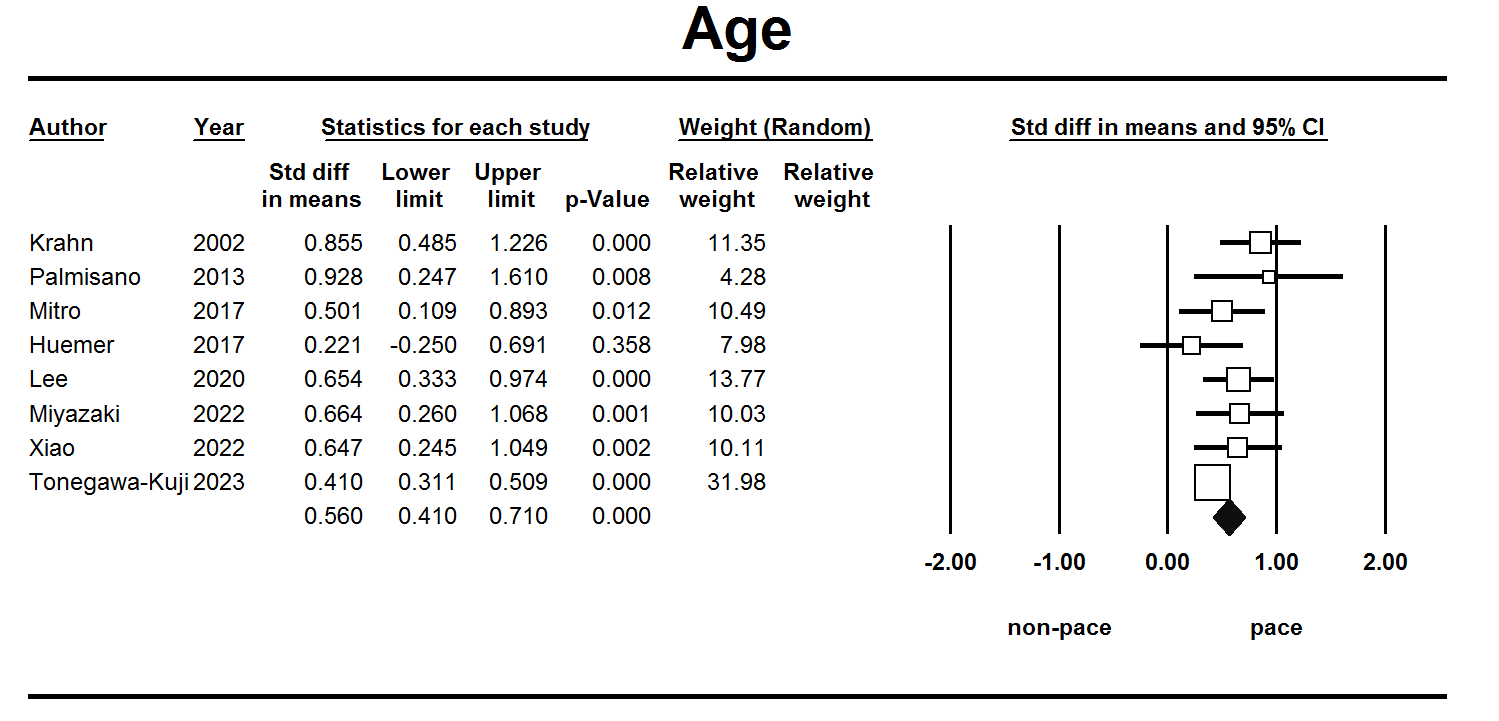


Supplementary figure 9. Standard mean difference of age between pace and non-pace groups.


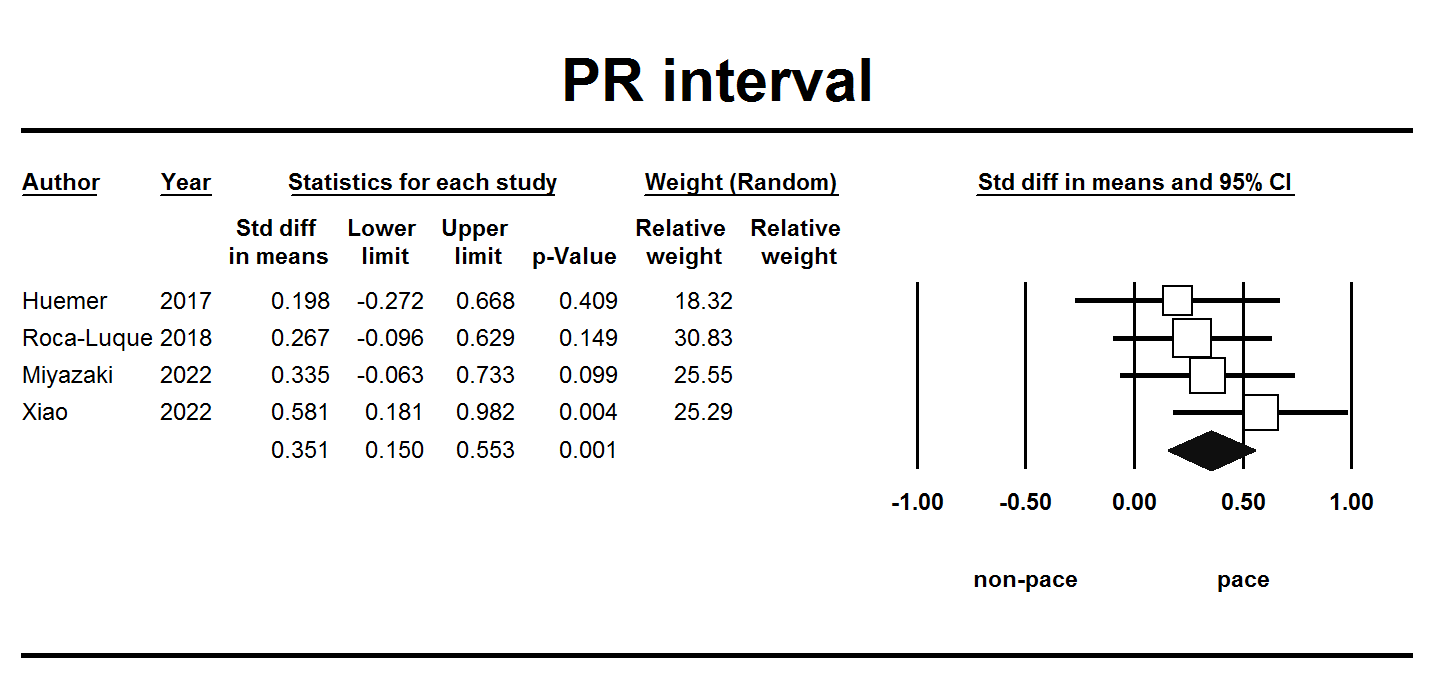


Supplementary figure 10. Standard mean difference of PR interval between pace and non-pace groups.


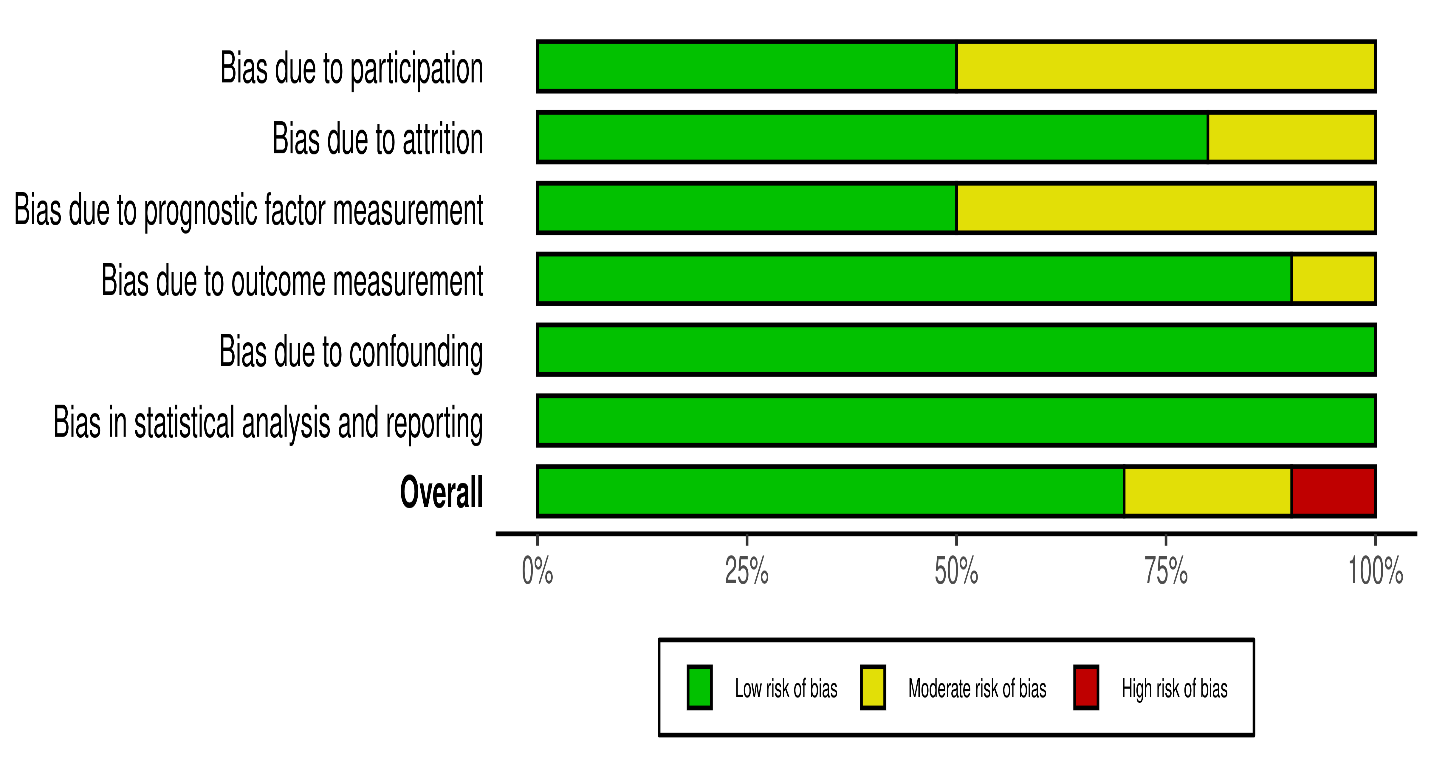


Supplementary figure 11. Summary bar plot of the distribution of risk-of-bias judgements within each bias domain.
