# Supplementary material for: Predictors of pacemaker requirement in patients with implantable loop recorder and unexplained syncope: A systematic review and meta‐analysis
Source: Clin Cardiol. 2024 Jan 29;47(2):e24221. doi: 10.1002/clc.24221 (PMC10823547; doi:10.1002/clc.24221)
Supplement: Supplementary file 3 — Supporting information. [file CLC-47-e24221-s001.docx]

Supplementary table 1. Duration and ECG findings of implanted ILR

| Author | duration of ILR | ECG findings while using ILR |
| --- | --- | --- |
| Krahn et al. | All patients completed at least 6 months of follow up | Tachyarrhythmic events including supraventicular tachycardia , Af, nonsustained venticular tachycardia, atrial flutter |
| Palmisano et al. | Median follow up duration was 279 days | New event Of tachyarrhythmia ,paroxysmal AF, supraventicular tachycardia, |
| Mitro et al. | The ILR remained implanted until a diagnostic event was recorded or until the end  of the battery life(follow-up of 9.4 ± 11.1 months) | Sinus pause (> 3 sec, < 6 sec),Sinus pause (> 6 sec),Sinus bradycardia, AF with slow ventricular response, SVT,AF with rapid ventricular response, Ventricular tachycardia, Ventricular fibrillation |
| Huemer et al. | Median ILR obsevation of 22 months (25th to 75th percentiles,12 to 28 months) | Sinus arrest >3 seconds (n=5); paroxysmal III-degree AV block (n=3); a period of 2:1 type II- degree AV block (n=1). prolonged asystole (6 sec) at the termination of an episode of atrial fibrillation (n=1); prolonged asystole (4 sec) during an episode of atrial fibrillation with low ventricular response (non-medicated patient) (n=1). |
| Roca-Luque et al. | 128 days(68_209) | Not reported |
| Lee et al. | meantime 233 ± 282 days | Sinus pause >3 s, AV block, Sinus bradycardia, Bradycardic atrial fibrillation, Sinus pause <3 s, SVT, nsVT <10 s/frequent VEB, Atrial fibrillation |
| Miyazaki et al. | median 81 ± 88 days | second-degree  AV block, third-degree  AV block, AF with a slow  ventricular rate, SVT, VT, sinus arrest |
| Xiao et al. | over 3 years(average time from ILR to PPM 213 days) | Atrial fibrillation, Left bundle branch block, Left anterior fascicular block, Right bundle branch block |
| Francisco-Pascual et al. | median of 3.4 years | Not reported |
| Tonegawa-Kuji et al. | follow-up of 27.9±10.8 months | Advances AV block |
